# Breast self-examination practice and associated factors among women aged 20–70 years attending public health institutions of Adwa town, North Ethiopia

**DOI:** 10.1186/s13104-018-3731-9

**Published:** 2018-08-29

**Authors:** Mebrahtu Abay, Gemechis Tuke, Eleni Zewdie, Teklehaymanot Huluf Abraha, Teklit Grum, Ermyas Brhane

**Affiliations:** 1 School of Public Health, College of Health Science, Aksum University, P.O.Box: 298, Aksum, Ethiopia; 2 Federal Ministry of Health, Addis Ababa, Ethiopia

**Keywords:** Breast self-examination, Practice, Women, 20–70 years, Adwa, Ethiopia

## Abstract

**Objective:**

Breast cancer is the leading cause of cancer mortality worldwide. The incidence of breast has been increasing in most regions of the world. Regular breast self-examination is one of the most cost-effective methods for early detection of breast cancer in asymptomatic women. Despite this fact, breast self-examination practice remains low in Ethiopia. Therefore, the aim of this study is to assess breast self-examination practice and associated factors among women aged 20–70 years attending public health institutions of Adwa town, North Ethiopia.

**Results:**

From the total study participants, only 26 (6.5%) of them had ever practice breast self-examination, and only 25 (6.25%) of them practice breast self-examination regularly. Being a government employee (AOR = 0.22, 95% CI = 0.071–0.683), having good perceived confidence to do breast self-examination (AOR = 5.32, 95% CI = 1.89–14.95) and having perceived good susceptibility to develop breast cancer (AOR = 3.79, 95% CI = 1.74–9.74) were the factors significantly associated with breast self-examination. Breast self-examination practice among the study participants was low. Therefore, informing every woman is susceptible to breast cancer, improving the confidence of women is recommended to increase breast self-examination practice.

## Introduction

Breast cancer is a type of malignant tumor which starts in the cells of the breast and commonly occurs in women than men [1]. Breast self-examination (BSE) is one of the screening methods which involves the woman herself looking at and feeling each breast for possible lumps, distortions or swelling [2].

Breast cancer incidence has been increasing in most regions of the world, but there are huge inequalities between rich and poor countries. Incidence rates remain highest in more developed regions, but due to lack of early detection and access to treatment facilities, mortality is much higher in less developed countries [3]. It is the most commonly diagnosed cancer in women, with an age-adjusted incidence rate of 28 per 100,000 women, and the second leading cause of death among women in Africa. The incidence varies across the continent, ranges from 19.3 per 100,000 women per year in eastern Africa to 38.1 per 100,000 women per year in southern and western Africa [4].

In Ethiopia, breast cancer is the second common cancer next to cervical cancer. It is estimated that around 10,000 Ethiopian women have breast cancer with thousands of more cases unreported as women living in rural areas often seek treatment from traditional healers before seeking help from health facilities [5]. It becomes fatal due to late presentation, limited resources, low awareness of breast cancer and its symptoms and strong traditional beliefs that can delay biomedical care [6].

Breast self-examination practice remains low in many countries. It was only 12% on the study in Kiewit [7]. Similarly, a study conducted in Nigeria showed that breast self-examination was only 18.1% [8]. BSE was also 28.1% in a study conducted in Debre Berhan town, Ethiopia [9].

Despite a lot of investment of government of Ethiopia in the health sector, communicable and infectious diseases are still the major health issues in the country. Cancer, particularly breast cancer, is on the bottom of the priority lists. That is why there is no much infrastructure and facilities to fight against breast cancer [10].

Although there are some early detection methods in health facilities of developing countries, they remain inaccessible to the women due to limited diagnostic and curative facilities. Mammography cannot be routinely applied in countries with limited health service resources since it is expensive it needs technology and trained professional [11]. Clinical Breast Examination (CBE) also needs professional skills and health facility visit to be conducted [7].

Breast self-examination is still recommended as a general approach to increase breast health awareness and allows for early detection of any abnormalities. BSE continues to be recommended by healthcare practitioners because it is free, simple, need low technology and teaching is possible [12].

Despite its prevalence, reproductive organ cancers (ROC) are not addressed as major public health problems at any level of the health care system. Nationwide, there is no organized ROC prevention, education, screening or curative care program [13]. Moreover, limited studies are conducted regarding BSE practice among women. The finding of this study will provide information to the concerned bodies (governmental and non-governmental organizations) to plan important interventions so as to improve women’s practice of breast self-examination. Therefore, the aim of this study was to assess breast self-examinations and associated factors among women aged 20–70 years attending public health institutions of Adwa town, northern Ethiopia.

## Main text

### Methods

#### Study design, study setting and population

A facility based cross-sectional study was conducted among women attending health facilities of Adwa town, North Ethiopia, from March 1st to April 15th, 2017. The town has two governmental health institutions; one health center and one general hospital. The health facilities had an average daily flow of 22 women aged 20–70 years. All women aged 20–70 years attending public health institutions of Adwa town were the source population, and systematically selected women were the study populations.

#### Sample size and sampling techniques

A sample size of 404 was calculated using single population proportion formula with assumptions of 39.4% prevalence of BSE practice [14], 5% margin of error, 95% confidence level and 10% non-response rate. All governmental health institutions found in the town were included in the study. The sample size in each public health institution was allocated proportional to the patient flow. Finally, systematic random sampling method was used to select participants from each institution.

#### Study variables and their measurement

The dependent variable is breast self-examination practice. Factors included in the model as independent variables are:

- Socio-demographic and economic factors like age, marital status, educational level, household monthly income, residence, employment and family history of breast cancer or cancer in other body parts.
- Knowledge, attitude and perception related factors like knowledge on breast self-examination, attitude towards breast self-examination and perception on breast self- examination.

Knowledgeable: participants who scored mean and above values from the provided 14 close-ended questions about the knowledge of BSE [15]
Good attitude: participants who scored mean and above values for attitude-related questions towards BSE, which was measured by the provided eight questions [15]
Perceived susceptibility: participants who scored mean and above values regarding their risk of breast cancer from the provided five close-ended questions were considered as having good perceived susceptibility [15]
Perceived benefits: participants who scored mean and above values regarding the benefit of BSE from the provided 6 close-endedquestions were considered as having good perceived benefits [15]
Perceived barriers: participants whose answered to barrier relate questions to do BSE measured by 6 questions, and scored mean above values were considered as having a perceived barrier to do BSE [15]
Perceived confidence: participants whose answered mean and above values regarding confidence to do BSE practice measured by 6 questions, were considered as having good perceived confidence to do BSE [15]

#### Data collection and data quality assurance methods

The questionnaire was prepared by reviewing relevant literature. Two days training was given to data collectors and supervisor on the aim of the study, the content of the questionnaire and on how to conduct the interview. A pre-test was done on 5% of the subjects in Aksum health center, found in Aksum town, Ethiopia. Data were collected using APRE-tested and interviewer-administered set of questionnaire. The collected data were cleaned and coded, and then entered into Epi-Info version 7 software and finally exported into SPSS version 21 statistical software for further analysis.

#### Data processing and analysis methods

Descriptive analysis was conducted and summarized using the mean, standard deviation for continuous variables and proportion for categorical variables. Before inclusion of predictors to multivariable logistic regression analysis, fulfillment of model assumption was checked. The goodness of model fitness was tested using Homers–Lemeshow test. Multi-co linearity was checked using Variance Inflation Factors (VIF).

Both bi-variable and multivariable logistic regression models were used to identify factors that affected BSE practice. Variables with a P-value of ≤ 0.2 in the bi-variable logistic regression model were entered into the multivariable logistic regression model. Variables with a P-value of < 0.05 in the final model were considered as independent factors associated with the BSE practice.

### Results

#### Socio-demographic characteristics of the study participants

Out of the expected 404 study participants, 400 of them give a complete response, with the response rate of 99%. About two-third, 266 (66.5%) of the study participants, were between the ages of 20–29 years. More than two-third, 276 (69%) of the study participants, were married. More than one-third, 156 (39%), of the study participants, were completed secondary education. More than one-fourth, 110 (27.5%), of study participants, were unemployed (Table 1).

**Table 1:** Socio-demographic and knowledge, attitude and perception on BSE characteristics of women attending public health facilities of Adwa Town, North Ethiopia, 2017

Variable	Category	Number (n)	Percent
Age group in years	20–29	266	66.5
	30–39	100	25.0
	40 and above	34	8.5
Marital status	Married	276	69.0
	Single	102	25.5
	Divorced, separated and widowed	22	5.5
Educational status	No formal education	34	8.5
	Primary education (1–8)	56	14.0
	Secondary education (9–12)	156	39.0
	Diploma and above	154	38.5
Employment status	Self employed	134	33.5
	Government employed	94	23.5
	Private employee	62	15.5
	Unemployed	110	27.5
Monthly income in Ethiopian birr	< 445	220	55.0
	445–1200	18	4.5
	1201–2500	20	5.0
	2501–3500	50	12.5
	3500 and above	92	23.0
Family history of breast cancer or any other cancer	Yes	10	2.5
	No	390	97.5
Knowledge about BSE	Knowledgeable	222	55.5
	Not knowledgeable	178	82.2
Attitude towards BSE	Good attitude	185	46.5
	Bad attitude	215	53.5
Perceived susceptibility	Yes	212	53.0
	No	188	47.0
Perceived benefit	Yes	172	43.0
	No	228	57.0
Perceived barrier to do BSE	Yes	168	42.0
	No	232	58.0
Perceived confidence to do BSE	Yes	248	62.0
	No	152	38.0

#### Knowledge and attitude of respondents on breast self-examination

To assess the knowledge level of the study participants, 14 close-ended questions were provided from those only 222 (55.5%), with 95% CI (50.8–61.0%), of them score mean and above value. An assessment of the participant’s knowledge of screening methods revealed that 246 (61.5%) participants know about breast cancer screening methods; from those 194 (48.5%) know types of the screening methods. One hundred and seventy-eight, 44.5% of the total respondents, had ever heard about BSE. Television was the predominant source of information to 100 (25%) respondents followed by health professionals 42 (10.5%) and radio 22 (5.5%). Less than half, 185 (46.3%) with 95% CI (41.5–51.5%), of the respondents had good attitude towards BSE (Table 1).

#### Perception of respondents on breast self-examination

More than half, 212 (53%), of the respondents perceived that they are susceptible to breast cancer. More than half, 212 (53%), of the respondents perceive that performing regular BSE is beneficial to find a lump in the breast, that might become cancerous in the future. One hundred and sixty-eight, 42%, of the respondents had perceived barriers to perform BSE, and 152 (38.0%) of the respondents were not confident enough to do BSE (Table 1).

#### Breast self-examination practice and factors associated with breast self-examination practice

About one-fourth, 26 (6.5%) with 95% CI (4.3–9.0%), of the study participants, had ever practiced BSE, and only 25 (6.25%) with 95% CI (4–8.6%), of the respondents practiced BSE regularly (Table 2).

**Table 2:** Breast self-examination practice among women attending public health facilities of Adwa town, Northern Ethiopia, 2017

Variable	Category	Number (n)	Percent
Had ever practice BSE	Yes	26	6.5
	No	374	93.5
Regularly practice BSE	Yes	25	6.25
	No	375	93.75
Frequency of practicing	Once in a week	7	1.75
	Once in a month	14	3.5
	Once in a 3 month	4	1.0
	When it comes to mind	1	0.25
Age started practicing BSE	< 25 years	9	2.25
	25–30 years	14	3.5
	30 years and above	3	0.75
When do you perform BSE	2–3 days after menses	16	4.0
	When it comes to mind	6	1.5
	Anytime during menses	3	0.75
	Regular day of each month	3	0.75
	Few days before menses	1	0.25
BSE practice in the last 12 months	1–3 times	7	1.75
	4 and above	13	3.25
	Did not perform in the last 12 month	6	1.5

In the multivariable logistic regression analysis, being a government employee, having good perceived confidence to do BSE, and having perceived good susceptibility to develop breast cancer, were the variables significantly associated with BSE practice. The odds of practicing BSE among self-employed women were 88% less likely compared to government-employed, [AOR = 0.22; 95% CI (0.071–0.683)]. Women who had a good perceived confidence to do breast self-examination were 5.32 times more likely to practice breast self-examination as compared to women who had low perceived confidence to do BSE, [AOR = 5.32; 95% CI (1.89–14.95)]. Women who had good perceived susceptibility to develop breast cancer were 3.79 more likely to practice BSE as compared to women who had low perceived susceptibility [AOR = 3.79; 95% CI (1.74–9.74)] (Table 3).

**Table 3:** Bi-variable and multivariable logistic regression of variables associated with BSE among women attending public health facilities of Adwa town, Northern Ethiopia, 2017

Variable	BSE practice		COR (95% CI)	AOR (95% CI)
	Yes (%)	No (%)		
Educational status				
Secondary education and below	9 (3.66)	237 (96.34)	1	1
Diploma and above	17 (11.8)	137 (88.2)	0.31 (0.133–0.71)	0.68 (0.26–1.81)
Employment status				
Self employed	5 (3.73)	129 (96.24)	1	1
Government employed	14 (14.89)	80 (85.11)	0.22 (0.077–0.638)	0.22 (0.071–0.683)*
NGO employed	6 (9.68)	56 (90.32)	0.362 (0.106–1.24)	0.284 (0.077–1.05)
Unemployed	1 (0.9)	109 (99.1)	4.22 (0.486–36.71)	1.68 (0.173–16.42)
Knowledge on BSE				
Knowledgeable	6 (2.7)	216 (97.3)	4.56 (1.79–11.61)	1.801 (0.633–5.12)
Not knowledgeable	20 (11.2)	158 (88.8)	1	1
Perceived susceptibility				
Good	8 (3.8)	204 (96.2)	2.7 (1.146–6.364)	3.79 (1.473–9.74)*
Low	18 (9.57)	170 (90.43)	1	1
Perceived confidence				
Good	6	242	6.11 (2.39–15.59)	5.32 (1.89–14.95)*
Low	20	132	1	1

* Significant at p-value < 0.05

### Discussion

According to the result of this study, only 6.5% of the study participants had ever practice breast self-examination. A similar finding was found in studies which were conducted in Kiewit and among female teachers of Kaffa Zone, Southern Ethiopia, both of them were 12% [7, 16]. But this study was found lower as compared to the studies done in Debre Birhan University, West Gojjam, which was 28.1% and 37% respectively [9, 17]. The difference in this study can be explained by the difference in the study participants. The participants in the above-mentioned studies were university students and health extension workers which could have a better understanding of the importance of breast self-examination than participants of this study.

In this study, only 6.25% of the respondents had regular breast self-examination. This is similar to the study conducted in logs, Nigeria, which was 11% [18]. But This result is lower as compared to studies done in west Gojjam and four zones of western Ethiopia, which were 14.4% and 33.7%, respectively [17, 19]. This difference could be due to differences of the study participants. The study participants in the mentioned studies were health extension workers and health professionals who are expected to have better knowledge on breast self-examination.

Self-employed women were 88% more likely to practice breast self-examination as compared to government-employed women. This can be due to the fact that self-employed women could have more time to check their breast and to do breast self-examination. Women who perceived as they are susceptible to breast cancer were 3.79 times more likely to practice breast self-examination as compared to their counterparts. A similar result was found in the study done Kaffa Zone, Ethiopia [16]. This could be explained by, women who perceived as they are susceptible to breast cancer may believe that breast self-examination could have a potential for early detection of a breast lump and to improve the outcome. The other explanation can be the perceived susceptibility to breast cancer may increase the perceived threat of respondents; hence they could practice breast self-examination more.

The odd of BES practice among women who had good perceived confidence were 5.32 times higher as compared to women who had low perceived confidence. A similar result was found in a study done in Iran among school teachers [20, 21]. This could be explained by respondents with good perceived confidence could have better motivation to practice breast self-examination.

## Limitation

The behavioral outcome is based on self-reported information. Therefore, the possibility of under or over-estimation may not be ruled out. Social desirability and recall biases may not be eliminated. It was not also triangulated with a qualitative method.

## Abbreviations

AOR: adjusted odds ratio
BSE: breast self-examination
CBE: clinical self-examination
CI: confidence interval
COR: crude odds ratio
CSA: Central Statistical Agency
ROC: reproductive organ cancer
SPSS: Statistical Package for Social Sciences
